# Barriers and Facilitators of Access to Psychological Services for Indigenous Populations: A Scoping Review and Thematic Analysis

**DOI:** 10.3389/fpsyt.2021.747054

**Published:** 2021-10-12

**Authors:** Anna Plessas, Moana W. Billot, Armon Tamatea, Oleg N. Medvedev, Jessica McCormack, Angelika Anderson

**Affiliations:** ^1^School of Psychology, Division of Arts, Law, Psychology, and Social Sciences, University of Waikato, Hamilton, New Zealand; ^2^National Institute for Health Innovation, Pacific Health, University of Auckland, Auckland, New Zealand

**Keywords:** indigenous, psychology, ABA, acceptability, barriers, facilitators

## Abstract

**Background:** The extent to which behavior-analytic interventions are offered to Indigenous populations across CANZUS in accessible and culturally appropriate ways is unknown. We conducted a scoping review with a thematic analysis of the extant literature to find: (1) what are the barriers and facilitators for providing effective and equitable delivery of psychological services (with a behavioral component) to Indigenous populations; and (2) what tools and practices exist for an effective and equitable service delivery.

**Methods:** We systematically reviewed Medline, CINAHL, PubMed, PsycInfo, Web of science, Ovid and INNZ databases between 1990 and 2020. For the scoping review, we adhered to the JBI methodological approach (2015) and the PRISMA strategy for the identification, selection, and appraisal of the reviewed articles. A total of 1265 unique articles met the criteria for the screening by title; 238 by abstract; 57 were included for full text assessment; and 37 were included in the final analysis.

**Results:** Three themes were revealed to account for the barriers and facilitators of culturally friendly practices: (1) connecting practices are about interactions shaping the relationship between service provider and service client; (2) innovative practices test new approaches and innovations that could facilitate access to psychological services and overcome barriers, and (3) reflective practices are about critically examining the processes and actions undertaken toward effective cultural adaptation of services.

**Conclusions:** Our analysis suggests that the level of success in bringing together services and the recipients of treatment (connection), showing flexibility and persistence in finding solutions (innovation) and examining the role of our behaviors in reaching our goals (reflection) is determined by the providers' action in the aforementioned three dimensions of practice.

## Introduction

Applied Behavior Analysis (ABA) is a scientific approach to improving socially important behaviors by the systematic application of the psychological principles of learning theory. Many of its applications are supported by a vast scientific evidence base. Though ABA-based interventions are most widely recognized as effective for improving developmental outcomes in individuals with autism spectrum disorder (ASD) (1), it has many other applications, including in healthcare, general education, gerontology, and business (2). Recently, the field of applied behavior analysis (ABA) has acknowledged its implementation of otherwise effective behavioral principles for behavior change as largely non-contextualized (3). However, behavior occurs in an environment (including culture); therefore, meaningful, lasting change in behavior occurs when the various environmental contingencies (including culture) impacting on behavior are accounted for and factored into a program of change.

The role of culture in shaping behavior has long been recognized in the field. For instance, Skinner characterized culture as a variable with multiple controls on behavior (4). ABA interventions are deeply rooted in a Western cultural context (5), and it appears that there is an established, one-sided standard of practice that disregards non-Western cultural variables as unnecessary to consider (6). A recent review of cultural adaptations to telehealth services revealed that most behavior analysts are challenged by the lack of relevant literature on specific cultural adaptations in relation to outcomes.

Indeed to date, no single study has explicitly evaluated or compared cultural adaptations for ABA-based interventions (7). Beaulieu, Addington and Almeida published a survey of behavior analysts that revealed that only 57% of behavior analysts work with diverse populations in the US (8). Further, they were reported to have little or no training in working with clients who present with marked cultural differences. The importance of culture in understanding social behavior—be it adaptive or maladaptive—is not a new issue in ABA (9, 10). The fact that behavior analysts fail to include cultural variables of behavior in explaining the social contingencies that control behavior may result in systematically overlooking the broader social context when designing and negotiating interventions. Arguably, the emphasis on essential learning principles, operationalizing observable behavior, empirical measurement, and adherence to laboratory-level standards of inquiry means that much nuance is removed from the understanding of behavior in ABA research and, in turn, practice. Given the call for cultural adaptations of ABA practice (11–13), this review is focused on the literature investigating barriers and facilitators for any psychological service that aims at behavior change.

Indigenous populations in Canada, Australia, New Zealand, and the United States (the CANZUS nations) are faced with unique challenges when it comes to effective and equitable delivery of services. The World Health Organization (WHO) defines equity in health as a process of minimizing factors of disparity—including, but not limited to, health care—between those from different social backgrounds (14). Many Indigenous peoples across CANZUS are over-represented with the burdens of sub-standard housing, poor education, unemployment, low income, cultural alienation, and other factors that contribute to the problem of health disparities. A recent review of the assessment of mental health services use by Indigenous populations across the CANZUS nations reported that only half of the surveys in their review had asked questions relating to interventions received and possible barriers to care (15). Their outcomes illustrate the fact that measuring service use by Indigenous populations has not been systematic and has not included indicators of the quality of the care delivered. The quality of services to Māori has recently been the focus of the New Zealand Health and Quality and Safety Commission, as inequity does not exist in access to services alone (16). For instance, people in New Zealand who live in socioeconomically deprived areas were 2.1 times more likely to experience psychological distress than other people living in less deprived areas. Māori (the Indigenous people of New Zealand) in particular fall behind non-Māori across all socioeconomic indicators, and they mostly live in under-resourced neighborhoods (14). Among other things, deprivation is a very serious barrier for those who need crucial services (17).

These observations are not explained by cultural difference *per se*. Many indigenous populations are uniquely disadvantaged as they find themselves in an involuntary culture contact situation as a result of having been colonized. When people from different cultures meet, various within- and between-group processes affect the developmental course of individuals and groups and their behavioral adaptations during the process of acculturation. Theories of acculturation (18–20) describe how involuntary culture contact situations can lead to maladaptive outcomes for minority groups and individuals, such as rejecting the values and practices of the mainstream culture, and thereby the keys to success within those cultures (21). Good outcomes of acculturation are promoted by positive attitudes toward both: one's own culture and the mainstream culture. It is therefore important to value and respect the cultures of individuals and to be able to work effectively with individuals who may have developed negative attitudes to the mainstream culture.

Though much has been written about the problems ethnic minorities have faced since the 1980s [e.g., (22)], only in the last decade newly published papers in North America have begun to inform clinicians on cultural adaptation, mainly for cognitive-behavioral therapy (CBT), whereas this matter has barely been investigated in other countries (23). For example, CBT has been adapted to treat refugees and ethnic minorities with anxiety or depression in the US, but cultural adaptation is still lacking for other psychological disorders (24). Evidence on the effectiveness of cultural adaptations is mixed and suggests that particular cultural adaptations may be beneficial for specific subgroups but not for others (25). A review of cultural adaptations for depression in minority ethnics highlighted that not all studies reported on what adaptations had been made; and the studies that did report this, were focused on the implementation of procedures rather than on the context of treatments (26). Moreover, a recent review of interventions for schizophrenia concluded that context, intervention, and design seem to influence the efficacy of cultural adaptations; however, adaptted interventions have not been compared to unadapted interventions (27). Nevertheless, cultural adaptations seem important if we are to promote acceptance of psychological interventions as a pre-requisite for effective and equitable delivery of services for those in need. Efforts to account for culture in interventions are a recent development (28), and cultural-behavioral systems scientists are increasingly contributing to a better understanding of the interaction between complex systems such as socioeconomic factors (29). In New Zealand, Te Tiriti o Waitangi puts an ongoing obligation on the Crown for partnership, participation and protection of Māori. As proposed in Plessas, McCormack and Kafantaris “for behavior analysts to achieve better outcomes, better accessibility and better choices of interventions for Māori, there is a need to develop skills that reflect cultural practices” [(30), p. 865].

This review aims to uncover and explore themes related to factors that impede or facilitate access to and participation in psychological services, as well as tools and practices to inform effective and equitable delivery of psychological services for Indigenous populations and in particular Māori. It is recognized that Indigenous peoples worldwide share common experiences of colonization and marginalization. In this paper, we focus on the issues for Māori, the “people of the land” (*Tangata whenua*), i.e., the Indigenous people of New Zealand, though we expanded our literature search to other Indigenous populations. In combination with considering the health system's obligations based on *Te Tiriti o Waitangi*, the results of this review will inform a survey methodology and will determine what questions a future survey should be asking. This scoping review aims to find answers to two main questions: (1) What are the barriers and facilitators for access to effective and equitable psychological services (with a behavioral component) for Indigenous populations? (2) What tools and practices exist in psychology (with a behavioral component) for an effective, culturally sensitive and equitable service delivery for Indigenous populations?

## Method

A systematic search was conducted to identify key themes in the literature regarding effective and equitable delivery of services to Indigenous populations –in particular, Māori– that may facilitate access and culturally appropriate service delivery.

The first step in the review process was the development of a study protocol by the first author that was reviewed by the research team, based on the guidelines provided by Joanna Briggs Institute Reviewers Manual on scoping review methodology (31). Following the guidelines, we defined from the outset our research questions, inclusion/exclusion criteria, search strategy and the study selection process for data extraction.

To answer our specific research questions, we conducted a scoping review with a reflective thematic analysis. We were particularly interested in finding out more about the accessibility and effectiveness of treatments specifically within behavior-analytic services, for indigenous populations, but it was not possible to identify such a narrow scope; therefore, we broadened our focus to extract data from all psychological services that target at least one behavior component for change. We included peer-reviewed studies and gray literature specifically for Māori. studies published from 1990 on. During the 90s, policies with a specific focus on improving Māori health were developed in New Zealand (32). Other CANZUS countries followed a similar trend regarding Indigenous cultures during that time; a search investigation showed that studies discussing cultural concerns started to be published around that time.

**Figure 1:**
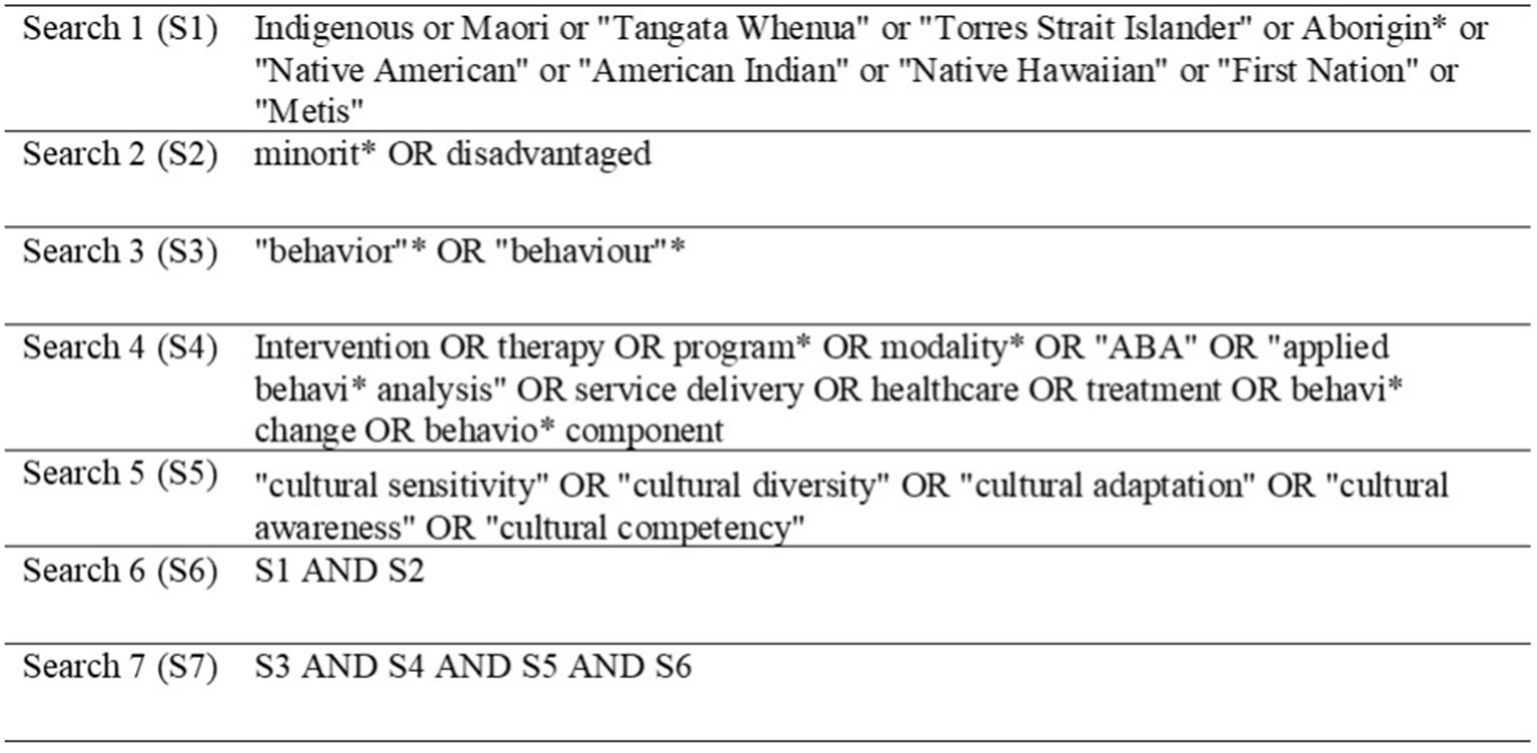
Search strategy.

Our search strategy was comprised of four steps The first author developed keywords (see Figure 1) and trained the second author to conduct the initial search with an expert librarian's support in psychological literature searches (Step 1). Six international databases (Medline, CINAHL, PubMed, PsycInfo, Web of science, Ovid), as well as the Index New Zealand (INNZ) database and all New Zealand journals, were searched between 1990 and 2020, and 1265 references were identified after duplicates were removed. We then carried on searching gray literature and the NZ Ministry of Health websites for government publications (Step 2). Twenty-four more articles were identified as relevant to our research question. After excluding all duplicates, using the bibliographic citation management software Endnote, we performed a manual check. This resulted in 1265 articles, which were screened by title (Step 3). Of these 785 were excluded as irrelevant to our questions, and further 285 were removed as they referred to non-Indigenous populations. The remaining 238 articles were reviewed by title and abstract (Step 4).

Our inclusion criteria were: (a) studies from CANZUS nations (Canada, Australia, New Zealand and the United States), (b) targeting Indigenous populations and reporting them as “disadvantaged” or “deprived” or studies targeting Māori (and Pasifika) regardless of socioeconomic status, (c) studies evaluating the accessibility and effectiveness of psychological interventions, or papers investigating tools and practices to facilitate culturally appropriate psychological service delivery or ways to improve services, (d) any study type (except prevalence and letters to editors) including discussion, evaluations, and reviews. We only included peer-reviewed literature, except for literature specifically related to psychological interventions or services for Māori.

The exclusion criteria were set as follows: (a) topics related to Organizational, Forensic or Health Psychology, (b) studies on sexual or physical education, criminal behavior, addictions, disease prevention, (c) studies on the participants' perceptions or feelings about health care providers *per se*, (d) studies related to health behavior (e.g., diabetes, contraception, etc.), (e) studies that discuss scale measures (i.e., validations), (f) studies that analyze cultural concept definitions (such as cultural competence or diversity) and (g) studies on ethnic minorities or social minority groups (e.g., LGBTQI+).

Our search was completed in March 2020, and an alert was set to notify the authors of any new studies meeting our search criteria that might be published until the end of 2020.

**Figure 2:**
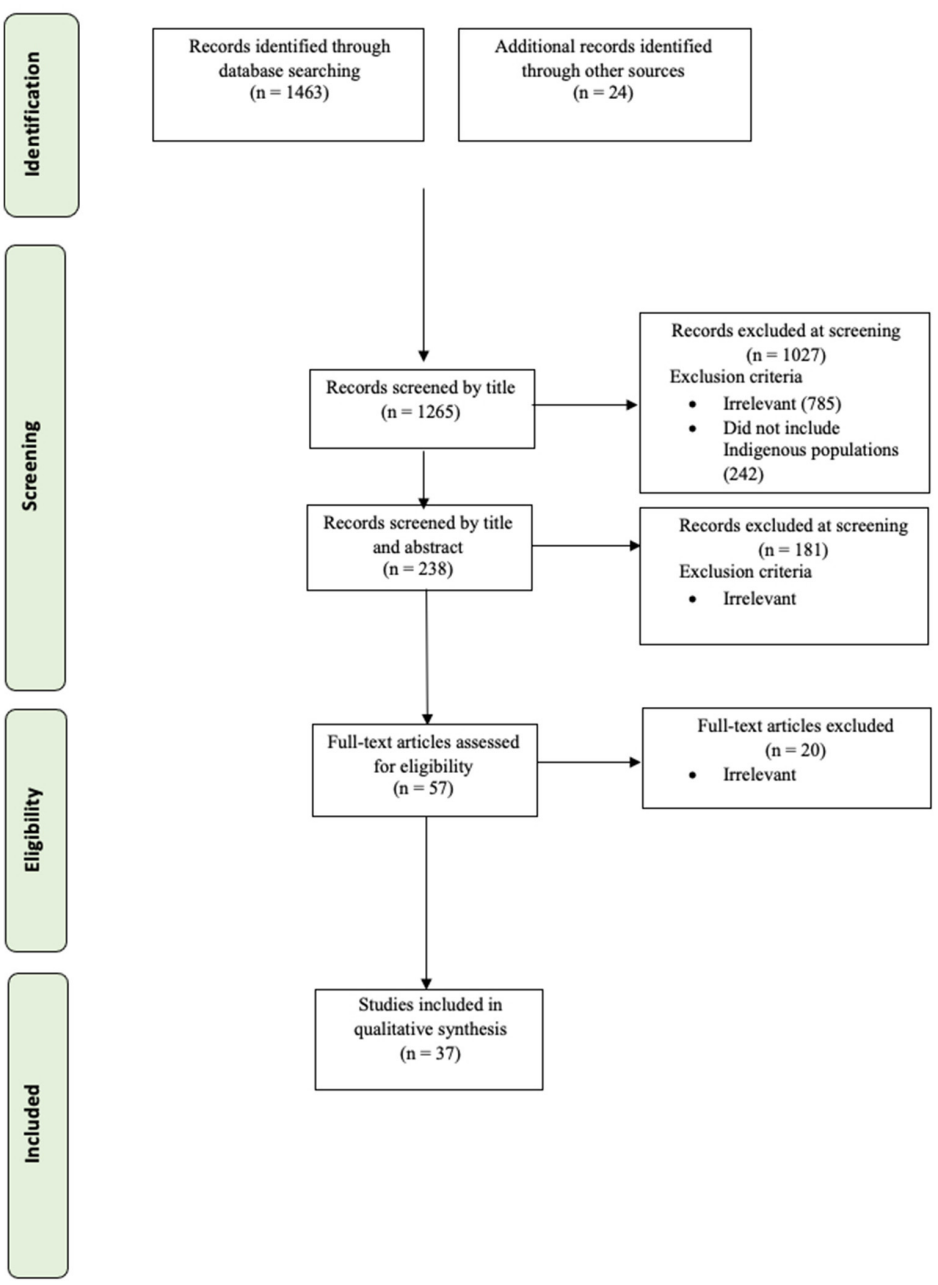
A PRISMA flow diagram showing the steps for the scoping review.

Both types of screenings, first by title and then by title and abstract, were conducted by three reviewers (AP, MB, JM). One of the reviewers was an Indigenous woman, while the other two reviewers were non-Indigenous. When titles only were screened, 25% (~300 articles) were reviewed independently by the second reviewer. Any time the reviewers were not certain, then the title was reviewed by at least two reviewers. Had any disagreements not been resolved between the first and second reviewers, an independent third reviewer (AA) would have determined the final inclusion. 51 articles from the research literature, and a further six articles from gray literature (=total 57 articles) met criteria for full article review. The first author conducted the full review, and ten articles were blind-screened by another author (JM). The full review led to the exclusion of the gray literature, except one, and 15 discussion and review articles. As a result, a total of 37 studies were included in the analysis. See Figure 2 for a flow chart of the PRISMA search strategy.

The data extracted were: Title, Authors, Year, Journal, Study type, Country, Service Type, Barriers and Facilitators, Tools and Practices, and suggestions for service provider training. The extracted data were analyzed through a reflective thematic analysis procedure using a theory-driven approach. The reflective thematic analysis procedure was conducted in an inductive way through six phases: (a) the first author familiarized herself with the data and highlighted and made notes on any text that was directly answering the two research questions, and (b) generated semantic (descriptive) codes from the extracted information to (c) construct themes; at first, (d) preliminary themes were constructed by the codes and were tested with JM and reviewed by all authors. Lastly, (e) the final definitions and themes were generated by telling a story of the data and (f) producing the results (33). Our research questions were guiding the analysis and we treated all the extracted information as a whole dataset. The approach used acknowledges that we are not separated from the data (34) and that the knowledge derived can be influenced by the diverse cultural backgrounds represented in our team and by our having different work experiences, including clinical and non-clinical experience. Our multinational team based in New Zealand includes professionals with a wide range of experiences in behavior analysis (e.g., education, mental health, mindfulness) and in other areas of psychology (i.e., clinical). The team is committed to contribute their fair share to the research into cultural adaptation to psychological practice. The composition of our team created a unique blend of perspectives on data and understanding of the patterns the data produced.

## Results

### Scoping Review Results

Table 1 summarizes the characteristics of the studies included in this review and Table 2 provides an enumeration and brief description of the details of each study. Ten of the 37 studies included an evaluation of a specific intervention regarding the cultural adaptations made during training; all studies described some sort of barriers and facilitators of psychological services (see Table 1 for an overview of the study characteristics). Most intervention studies involved parent training programs (Studies 1, 7, 16, 18, 29, 33, 37) and cognitive-behavioral therapy (Studies 2-5, 17, 22), though several others were based on cognitive-behavioral principles. Only one paper employed single-case research methods to assess the behavioral impact of an intervention (6). The remaining studies employed mainly qualitative methods save two studies that used quantitative methods (Studies 14-16). Several Indigenous populations were included: First American Indians/Alaska Natives (Studies 5-6, 8-10, 12, 15, 18-19, 25-26, 31, 35), followed by Māori (Studies 2-3, 7, 11, 14, 16, 21-22, 27-28, 32), followed by Aboriginal and Torres Islanders (Studies 1, 4, 13, 17, 29, 33, 36, 37). A few studies included Aboriginal Canadians and First Nations (Studies 11, 20, 23-24), one study included Pasifika (Study 34), and another one Inupiat Eskimo (Study 30). Fourteen studies were from the USA (Study 5-6, 8-10, 12, 15, 18-19, 25-26, 30-31, 35), 11 were from New Zealand (Study 2-3, 7, 14, 16, 21-22, 27-28, 32, 34), eight from Australia (Study 1, 4, 13, 17, 29, 33, 36-37) and four from Canada (Study 11, 20, 23-24).

**Table 1:** Characteristics of the study.

	**Variables**	***n***
Countries	USA	14
	New Zealand	11
	Australia	8
	Canada	4
Type of papers	Evaluation of interventions: (total = 10)	
	a) Qualitative Measures b) Quantitative Measures of Intervention	8 2
	Review Studies	7
	Discussion Paper (ABA = 1)	7
	Feedback post-interventions	6
	Codesign (pre-delivery of any service)	3
	Cross sectional survey	2
	Participatory action research group	1
	Supervisory tool description	1
Indigenous population	American Indians/Alaska Natives	13
	Māori	11
	Aborigines and Torres Strait Islanders	8
	Canadian first nations and inuit	6
	Pasifika	1
Intervention characteristics	CBT (Culturally adapted)	6
	Other parent training interventions	5
	School positive behavior plans	3
	Triple P parent training programme	2
	Home intervention	1
	Treatment packages	1
	Behavior management	1
	Psychotherapy	1
	Mental heath first aid	1

**Table 2:** Summary of extracted data by source for this review.

**Study**	**1st Authors/Publication Year/Study Type**	**Country and Indigenous Group**	**Service Type**	**Facilitators**	**Barriers**	**Tools/practices**	**Suggestions for Service Provider Training**
S1	Andersson et al. (35) Study Protocol for Intervention	Australia. Aborigines and Torres Strait Islanders.	Triple P parenting programme	Flexible to the structure of the programme.	Reducing barriers to attendance by providing transport, childcare, and meals.	Deliver programme in a culturally meaningful manner, train participants to become coaches of the programme.	
S2	Bennett et al. (36) Applied Research	New Zealand. Māori.	CBT	Culturally adapted CBT for Māori. Process for engagement, spirituality, family, and metaphor use.			
S3	Bennett et al. (23) Case Studies	New Zealand. Māori.	CBT	Adaptations of practice has been organized into four domains: connectedness, spirituality, extended family, and metaphor.	Evidence-based practice does not involve spirituality. Limited research on the evolving nature of Māori culture.	Build relationships, develop culturally adapted materials, schedules that promote enjoyment and engagement.	
S4	Bennett-Levy et al. (37) Qualitative	Australia. Aborigines and Torres Strait Islanders.	CBT	Adaptations to CBT to fit different social and cultural contexts.		Using a variety of methods for presentation of therapies.	Using CBT and being guided by their clients and their own estimations about its suitability and usefulness.
S5	BigFoot et al. (38) Discussion	USA. American Indians/Alaska Natives.	CBT	The adaptation of TF-CBT within an American Indians/Alaska Natives well-being framework.	The assumption that all Indigenous people value or practice indigenous concepts or tribal beliefs the same way.	Culturally adapting language and core principles with treatment concepts.	Supervision and ongoing consultation.
S6	Chomos et al. (39) SSD	USA. American Indians/Alaska Natives.	Home visitor's treatment	Increase cultural knowledge and practices. Incorporating cultural enhancements: sharing of stories and information on traditional foods and medicine, smudging.		Using Single Subject Design (SSD) to evaluate cultural enhancements in a variety of settings-important for small tribal communities. SSD allow everyone to be involved and understand the results, can be sensitive to culture.	
S7	Cope (40) Narrative	New Zealand. Māori.	Parent Training	Group leaders learn to promote collaboration through reflection, reframing, reinforcing, support and acceptance of parent perceptions and ideas.		Cultural advisors using Māori procedure/protocol and framework during therapies.	
S8	Gameon et al. (41) Systematic Review	USA. American Indians/Alaska Natives.	N/A	Culturally- grounded interventions. Delivering surface-level adaptations of trauma treatment.	High rates of trauma in Native communities and only a small number of intervention programmes available.	Using a culturally grounded approach. Researchers working with communities to adapt interventions for future use.	
S9	Gone et al. (42) Review	USA. American Indians/Alaska Natives.	N/A	Increase funding. Culturally grounded interventions.	Lack of American Indians/Alaska Natives psychiatrists. Scarce evidence-based treatments administered. Insufficient allocation of funding. Frequent provider turnover. Favoring pharmacotherapy over psychotherapy.	Help seeking for the AI-SUPERPFP tribal populations from mental health professionals, other medical professionals, or traditional healers.	
S10	Goodkind et al. (43) Review	USA. American Indians/Alaska Natives.	N/A	Funding cultural teachers.			
S11	Gutierrez et al. (44) Review	Canada. Aboriginal Canadians, Māori.	N/A	Integrating traditional healing approaches with CBT.	Services not targeting needs in a culturally responsive manner.	Services based on traditional Māori cultural rules or ways of life. Creation of specialized roles for Māori staff. In-house cultural sensitivity training.	
S12	Haozous et al. (45) Focus Groups	USA. American Indians/Alaska Natives.	CBPM	Telehealth. Culturally knowledgeable providers.	Patient reports of being ignored, undertreated, disregarded or overlooked. Funding barriers.	Using traditional American Indians/Alaska Natives activities and herbal remedies. Having clear communication.	
S13	Hart et al. (46) Mixed Methods	Australia. Aborigines and Torres Strait Islanders.	Mental Health First Aid	Being aware of relevant cultural factors in mental illness and the need to facilitate supporting relationships when delivering first aid.	Poor mental health literacy for minorities.		
S14	Hatcher et al. (47) Zelen RCT	New Zealand. Māori.	Treatment Package	Cultural treatment package.	Need to find what cultural aspects encourage engagement.		
S15	Johnson et al. (48) Theoretical	USA. American Indians.	N/A		Limited rural services and use of alternative therapies. Patient/clinician come from different cultural backgrounds.	Using interpreters.	
S16	Keown et al. (49) RCT	New Zealand. Māori.	Triple P parenting programme	Collaborative Participation Adaptation Model to culturally adapt a programme. Brief, effective, culturally adapted parenting support is visible and accessible to Māori families.		Using elders/experts in community to assist practitioners to be culturally appropriate.	
S17	Killcullen et al. (50) Qualitative	Australia. Aborigines and Torres Strait Islanders.	CBT	Build cross-cultural understanding. Using a cognitive and behavioral framework within the social-emotional well-being model provides culturally appropriate reference points for working at the cultural interface.		Using practical behavioral skills and mindfulness meditation.	Understanding both the points of similarity and divergence in perspective of mental health across cultures.
S18	Kulis et al. (51) Descriptive Statistics	USA. Urban American Indians.	Parent training	Parenting 2 Worlds application improved cultural engagement AI–cultural elements that resonated across tribal heritages.	Need for evidence-based and culturally grounded parenting interventions.	Workshops where participants actively share knowledge and experience of AI cultural values.	
S19	Lewis et al. (52) Review	USA. American Indians/Alaska Natives/First Nations.	N/A	Providers learned culturally appropriate interpersonal behaviors and changed their practice style.	Social determinants of health. No incorporation of Indigenous health beliefs. Providers have no training.	(1) Financial and administrative considerations, (2) Shared theoretical foundation, (3) Family and community, (4) Patient- centered care, (5) Interprofessional, (6) Flattening the patient provider hierarchy. (7) Interdisciplinary training.	
S20	Lints-Martindale et al. (53) Review	Canada. Aboriginal Canadians.	N/A	Telehealth. Rebuilding respectful relationships. Incorporate Indigenous healing practices in strategic frameworks and programmes.	Putting recommendations into practice.		Cultural diversity training.
S21	Lipsham (54) Narrative	New Zealand. Māori.	Māori Model	Developing a Māori thinking through the supervision model Āta.		Āta as a tool.	
S22	Mathieson et al. (55) Qualitative.	New Zealand. Māori.	CBT	A culturally appropriate collaborative approach to intervention adaptation can result in a talking therapy.	Research on adapting talking therapies for Māori is scant.	Resources showed an increased emphasis on forming a relationship; spirituality; increased use of Māori language and changes to imagery in self-management booklets.	
S23	McCabe (56) Qualitative	Canada. Naive Americans and Aboriginal Canadians.	Psychotherapy	Traditional treatment model intervention in a psychotherapy framework/community-derived and culture-based therapy model.	Lack of success in overcoming cultural disconnections. Limited availability and underused services,	Increasing acceptance to change and discover self, trust in service and healing spirit.	Biculturalism.
S24	McIntosh et al. (57) Case Studies	Canada. Aboriginal Canadians.	School-Wide PBIS	Schools being culturally responsive.	Cultural disconnect and a mismatch between school expectations and cultural values.	Conceptualizing problem behavior within the cultural context. Understanding cultural values to inform shared goals. Cross-cultural competence for overall design of acceptable interventions.	Efforts must be made to understand Indigenous students and their histories on a deeper level.
S25	Morsette et al. (58) Mixed Methods	USA. American Indians.	School-Wide CBITS	Encouraging school staff to invite Native Elders and healers to participate in the initial CBITS treatment session and the final “graduation” ceremony. No records of those activities were collected or disclosed to outsiders.	The partial disclosure of traumatic events (avoid trauma due to violence).		Refer to a cultural advisor when spiritual experiences are shared, and questions are asked.
S26	Pina et al. (59) Systematic Review	USA. Native American.	N/A	EBI and resources should be accessible to practitioners and training providers. Practitioners holding culturally informed views of mental health problems.	Lack of adequate sample sizes of different ethnicities. Lack of theoretical rationale.	High quality training, monitoring and technical assistance, and disclosures about costs.	
S27	Plessas et al. (30) Theoretical	New Zealand. Māori.	N/A	Ensuring practitioners have the skills to work with Māori. Measuring clinical performance against the cultural needs of Māori. Developing interventions with Māori.			Cultural training and upskilling to ensure practitioner's competence.
S28	Rangihuna et al. (60) Discussion	New Zealand. Māori.	Māori Model	Rapid development of therapeutic relationships, the identification of the “problem” through a Māori lens, the injection of meaning into the pathway ahead, and the sharing of a common set of understood values, beliefs and practices.		Offering an alternative way (Māori narratives) to frame withdrawal, outrageous behavior etc. to reflect upon their own situation and to develop a pathway for a therapeutic intervention.	Train professionals how to use the approach.
S29	Robinson et al. (61) Statistical Analysis	Australia. Aborigines.	Parent training	Systematic attention to cultural “fit” of the intervention. Logic and cultural competence in engagement of disadvantaged families.	A combination of sociodemographic and other factors influencing the measurement of children's perceived behaviors.		
S30	Sexton et al. (62) Theoretical	USA. Inupiat Eskimo.	Counseling	Applying cultural competency, Building Trust.		Building rapport with village, being flexible and creative in delivering therapy sessions.	Seeking ongoing professional supervision.
S31	Snowden et al. (63) Theoretical	USA. Minorities/Native Americans.	N/A	Well-founded understanding is important to guide outreach programming, incentive management strategies, program design efforts, and increased practitioner awareness.	The MH structure produce wide-range variations in rates of access. We lack to adequately explain the lack of access.		
S32	Stead. (64) Theoretical	New Zealand. Māori.	N/A	Practicing cultural responsiveness.	Education of psychologists have no access to cultural advisors.		Actively engaging and showing integrity, sincerity and respect toward Maori beliefs, language and culture.
S33	Stock et al. (65) Qualitative	Australia. Aborigines.	Parent training	Two-way trust and respect, learn and understand each other, reflective practice. Potential accreditation of Aboriginal staff in their communities and building capacity and programme sustainability.	Centrality of friendship to successful relationships between team members raised critical questions about professional boundaries.	Time to build rapport and develop mutual mentoring programmes.	
S34	Tauai et al. (66) Survey	New Zealand. Pasifika.	N/A	Pacific languages have a protective effect against common mental disorders.	Need for appropriate translation services and/or Pacific language speaking mental health professionals.		
S35	Tsosie et al. (67) Qualitative	USA. American Indians/Alaska Natives.	Management treatment	Integrate evidence-based interventions and traditional Native healing into care treatment programs. Reconnect patients to Native culture.		American Indians/Alaska Natives case manager. Culturally tailored intervention.	
S36	Turner et al. (68) Online Survey	Australia. Aborigines.	Professional training for Indigenous practitioners.	Support for organizations to develop appropriate supervision and training resources.	Evidence-based programmes by Indigenous practitioners are largely untested. Limited professional development training courses.	Comfortable training environments that include *in-vivo* training, practice, goal setting and self-evaluation.	Professional training for Indigenous practitioners.
S37	Wagner et al. (69) Mixed Methods	Australia. Aborigines.	Parent training	Adjusted programme to honor the cultural and linguistic diversity.		Programme piloted in relevant cultural setting.	

*N/A, not applicable; CBT, cognitive-behavioral therapy; TF-CBT, Trauma focused CBT; CBPM, CBT for pain management; PBIS, positive behavioral interventions and supports; CBITS, CBT for trauma in schools; AI-SUPERPFP, American Indian Service Utilization, Psychiatric Epidemiology, Risk and Protective Factors Project*.

### Thematic Analysis

**Figure 3:**
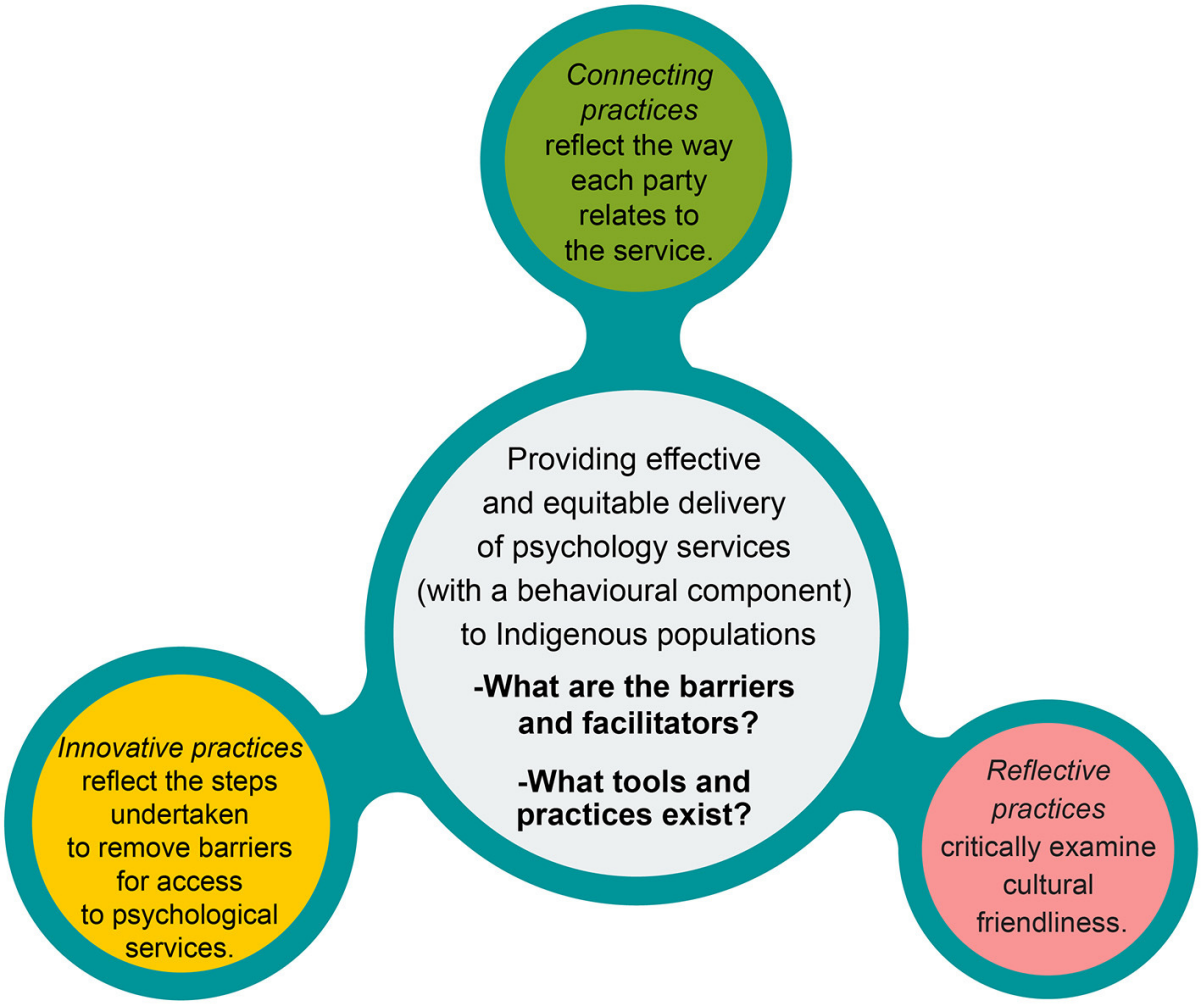
A schematic representation of the developed thematic map.

The information was categorized according to our research questions into three main areas: (1) barriers related to overall implementation issues of psychological interventions for Indigenous populations and, in particular, to the accessibility of services; (2) factors/variables that facilitate cultural adaptations during the discussion or implementation of interventions; and (3) suggestions on specific tools and practices, or service provider training recommendations, that have been introduced or discussed as possible steps for improvement. We generated three overarching themes to account for the barriers and facilitators of cultural practices. Each theme revolved around a central organizing concept (see Figure 3) that provided a description of the theme data: (1) *connecting practic*es are about interactions shaping the relationship between service provider and service client; (2) *innovative practices* test new approaches that could facilitate access to psychological services and remove barriers, and (3) *reflective practices* are about critically examining the processes and actions undertaken toward effective cultural adaptation of services. These themes are distinct in the way the barriers and facilitators are approached but can overlap in regard to variables/factors. For example, under “innovative practices,” a barrier factor may be approached by testing novel strategies through trial and error aiming at removing the barrier, while under “reflective practices” the same barrier can be approached critically by investigating actions that have contributed to it. At the same time, and in spite of the barrier, “connecting practices” may support an ongoing relationship between service and client. Table 3 summarizes the subthemes related to the overarching themes.

**Table 3:** Summary of themes and subthemes that emerged from thematic analysis.

**Overarching theme**	**Subtheme**	**Description**
Connecting practices	1. Relationship building	•The pre-disposition, or experience of not having a sense of relationship security, comfort, or partnership •Any action taken toward building a positive relationship between the treatment provider and the treatment recipient •Any specific action to build relationships
	2. Engagement	•Do not engage in behaviors that will encourage engagement. There is no encouragement to involve anyone from the extended family or the community •Involving people that promote engagement in all stages of the treatment plan •Any action that promotes engagement on all levels during the treatment process
	3. Diverse views	•Level of congruency between Indigenous and western world views that lead to different expectations, values etc. •Any action taken to include indigenous knowledge in the making of interventions and application
	4. Collaborations	•The level of collaboration on all stages of treatment or research process •Any action taken toward working jointly to achieve culturally fit interventions that indigenous populations and treatment providers will be willing to engage in order to achieve positive outcomes
	5. Sense of ownership	•A lack of services on a local level that not only are available locally but share the same culture •Any action taken to minimize disparity between indigenous and non-indigenous individuals involved in treatment
Innovative practices	1. Adapting interventions	•Either inability or absence of effort to adapt interventions to clients' needs within cultural context •Evidence based research does not respond to cultural considerations regarding Indigenous populations •Interventions are adapted to fit the needs of indigenous populations in a cultural context •Approaching treatments through practical solutions and through the best available current research
	2. Resources toward adapting Interventions	•Any tools either during the assessment stage or the implementation phase that facilitate cultural acceptance and inclusion of those factors that promote positive treatment outcomes •Creating or providing explicit materials that promote cultural acknowledgment
	3. Innovative training approaches	•Any education that promotes cultural practice •Adapt one part of the educational curriculum to develop skills in relation to cultural practices of diverse backgrounds •The inclusion of teaching theoretical alternative models and cultures across departments •Depart from standard/stereotyped practices when teaching cultural practices •Any action taken by the profession after completion of studies to keep culturally adapted practice up to date
	4. Recruitment	•The recruitment and training of diverse populations. Creating a workforce
Reflective practices	1. Reflective scope	•Building awareness and openness to other people's cultural beliefs and building the competency to be a valuable member in a diverse relationship
	2. Practical limitations	•Treatment is not accessible because of barriers related to practical concerns that need to be considered in order to reach the appointment site or to adhere to the treatment
	3. Attitudes toward treatment	•Negative pre-dispositions toward the outcome of treatment that are related to personal experiences or views of mental illness
	4. Limited options	•Limited knowledge or access treatments available, accessing only particular types of therapies
	5. Unwillingness/bias	•The institution or practitioner providing excuses for the unavailability of treatment options •The assumption for or against one person (indigenous and non-indigenous individuals involved in treatment)
	6. Language barriers	•Different usage of language as modality and lack of means that can resolve this matter

#### Connecting Practices

Connecting practices are defined as any action that can move clients and service providers closer or apart depending on the action taken by the treatment provider or the treatment recipient. The results suggest that the ability or inability to create a relationship from the start, and to sustain this throughout the time of service provision is crucial. Clients often come in with a sense of guarded anticipation, or experiences of insecure relationships, or a lack of comfort or partnership. Action needs to be taken to build relationships. For example, the literature reports issues regarding confidentiality, distrust on the part of diverse populations, or partial disclosure of what may be the referral problem (as in Study 7). However, if professionals make sure effective communication is in place, build working relationships with elders in the community, even participate in Indigenous ceremonies, then by getting to know each other, relationship-building hurdles can be overcome.

The level of engagement may interfere with the relationship, even before accessing the service. For example, studies report that those in need for intervention do not always initiate help-seeking behaviors or may show an unwillingness to use the service (Study 15). However, outcomes can be promising when one involves those promoting engagement at all stages of the treatment (e.g., peer counselors, family intervenors or other indigenous specialized roles) or when action is taken toward that direction by including the whole family and providing community support. For connecting practices, we also included the level of congruence between western worldviews (expectations, values, etc.) and actions taken to include indigenous knowledge in the design and implementation of treatments by having an indigenous methodology in the treatment plan or by integrating alternative treatments.

Further, the literature indicates that it is also critical to consider the level of collaborations at all stages of a treatment or a research project (e.g., consent vs. compulsory treatment) with actions taken toward working jointly to achieve a culturally fit intervention. An example of a positive collaboration is funding to incorporate indigenous practices by paying for traditional practitioners for their service (Study 10) and aligning this involvement with evidence-based practice (Study 35). Lastly, an essential element to connecting practices is the sense of ownership, meaning that the practitioner shares a similar culture with the client and is a local presence in the community. This for example can be achieved by taking actions that minimize disparities between indigenous and non-indigenous individuals involved in the treatment (as in Study 37).

#### Innovative Practices

Innovative practices are defined as the level of flexibility shown to fit interventions or training programs to cultural practices by adapting them and developing creative training opportunities. Innovative practices need to consider how interventions are adapted to fit indigenous populations' needs in a cultural context and the number of practical solutions provided based on the best available current research. For example, the literature reports that interventions often fail to take into account indigenous behaviors that affect engagement in the intervention. At the same time, the evidence suggests that culture-tailored interventions should not be regarded as a one-size-fit-all treatment (as in Study 11); however, more often than not, interventions are not culture-responsive, as there is a lack of literature on cultural adaptations (as in Study 22) or behavioral measurements of adaptation (as in Study 26). It seems reasonable to consider a shift to practice-based evidence and use more detailed reports on the individual client's demographics when applying an intervention. The tools used either during the assessment or the implementation phase are part of the process; they can facilitate cultural acceptance and inclusion of factors that promote positive treatment outcomes, such as the conceptualization of a problem behavior in its cultural context. Resources that enable cultural acknowledgment, by adapting traditional names for core concepts for example, can be beneficial too.

Any education that promotes cultural practices on any level is critical to psychology practices. A few examples from the literature are to conduct workshops that target sharing knowledge (mutual-way mentoring) (Study 33), to provide education to policymakers (as in Study 27), to train providers in adapting educational curricula to develop cultural practice skills of diverse backgrounds (Study 36), to teach alternative theoretical models (i.e., indigenous thinking) and cultures across university departments (e.g., Study 28). These suggestions are accompanied by innovative teaching strategies that depart from standard methods of teaching cultural practices, not to mention the recruitment and training of diverse populations for the future workforce. Even after graduation, ongoing work is necessary, with cultural advisors, peer support and mentoring being regarded as tools to keep service providers aligned with culturally adapted practices.

#### Reflective Practices

Reflective practices are about critically examining positive and negative behaviors in the diverse therapeutic relationship, as well as the processes and actions undertaken toward effective cultural adaptation of services. The literature reports that treatment may not be accessible due to barriers related to practical difficulties (as in Study 1), such as traveling to the appointment site or adhering to treatment (e.g., transportation, childcare assistance, other social determinants of health); when the aim is to provide accessible services, such knowledge needs to be critically reflected upon. Moreover, one has to be aware of clients' negative anticipations regarding the outcome of treatment based on their personal experiences or views on mental health, such as their beliefs about the diagnosis, lack of trust or the stigma that may be associated with the whole process. It has also been reported that indigenous populations seem to have limited knowledge of or access to available treatments as they are only accessing specific types of therapies; for example, they may be unfamiliar with ABA services' availability.

Indigenous populations have reported that treatment providers often provide excuses regarding the unavailability of treatment options (Study 31). Biases for or against indigenous or non-indigenous populations have been reported; for example, the existence of a western bias (Study 24) or the lack of recognition or under-estimation of cultural differences (Study 5). Lastly, reflective practices require considering the different usage of language as a modality (e.g., cultural connotations) and sourcing the means that can resolve the matter (e.g., access to interpreters). Building awareness and openness to other people's cultural beliefs and building the competency to be part of a diverse relationship (e.g., seeking ongoing feedback and needs in the process of cultural adaptation) is a core pre-requisite of success in this endeavor.

## Discussion

The aim of this scoping review was to explore barriers and facilitators for access and participation in psychological services with a behavioral component (e.g., ABA services), as well as tools and practices for effective and equitable delivery of psychological services for Māori and other Indigenous populations. This scoping review revealed that the way therapists' practice may determine the level of opportunities provided for effective and equitable services by either practitioners or organizations/institutions they are representing. Our thematic analysis organized the information extracted from the literature around three key elements that expanded our understanding of barriers and facilitators: connection; innovation; and reflection. The analysis suggests that successful culturally appropriate practice may be facilitated by bringing together our practice and the recipients of interventions (connection), showing flexibility and persistence to find solutions (innovation), and evaluating the role of our own behaviors in this context (reflection). In summary, our findings aligned with current knowledge from across the health, disability and welfare sectors: disparities in the access to services and in the quality of services are apparent between Indigenous and non-Indigenous service users in all CANZUS countries. Therefore, our findings contribute to the existing body of research into cultural adaptations of psychological and behavioral interventions e.g., (11–13, 22) and highlight the importance of cultural adaptations and their specific benefits to ABA-based interventions across a wide range of cultures and countries. Thus, this review may also encourage professionals worldwide to identify barriers for such adaptations and inform strategies to overcome such barriers.

In applying theories and practices of psychology, any psychological service that is committed to behavior change has to adhere to the technology of behavior (10). ABA research predominantly bases therapy on the understanding of learning mechanisms and behavior change. However, the scarce number of published papers in the ABA literature suggests that behavior analyst have given little attention to the effect of cultural variables. One possible explanation for the paucity of relevant ABA literature could be the challenge of defining or classifying behaviors or practices as “culturally adapted” in measurable terms or the challenge of investigating how such behaviors are acquired. Take for example a Māori client who always arrives late or skips sessions because s/he is too embarrassed to let the practitioner know that s/he cannot afford to take leave from work. In this example, which behaviors or practices could a therapist adopt that can be classified as “culturally appropriate” to promote accessibility?

Outside the cultural context of the anglophone world, behavior analysts often use ethnic cultural behaviors to facilitate access to services. However, there has been no research investigating how individual ABA practitioners adapt their approaches and interventions in their respective cultural contexts, nor have such practices been highlighted in individual case studies. This lack of reports on cultural adaptations is observed across all schools of approaches to psychological intervention (15). Opening a discussion about research into how cultural practices can enhance the accessibility and effectiveness of interventions could be the pathway toward a better understanding of potential outcomes of cultural adaptations.

Asking such wh- questions could become a helpful tool for effective delivery of psychological services based on the three themes (connection, innovation, reflection) that emerged from our thematic analysis. It can generate further questions (reflective practice) or even put a measurement system in place for behaviors that contribute to cultural adaptation (innovative practice). Another potentially useful application of this review's findings could be for the psychologist or behavior analyst to consider what behaviors (connecting practices) have assisted their client to stay in therapy and allowed the client to decide what works best for them. Such an approach could promote our understanding of who cultural adaptations are suitable for, as cultural adaptations cannot come as a “one-size-fits-all.” Each Indigenous individual may identify with a specific subgroup but also has unique experiences from living in a westernized world. The literature reports mixed outcomes e.g., (25) of efficacy and effectiveness of current culturally adapted practices and this scoping review is adding its bit in the direction of client-tailored, individualized approaches.

The importance of cultural adaptation to psychological practices cannot be overstated. In recent years, professional psychological associations (e.g., the American Psychological Association, the British Psychological Society, the Australian Psychological Society, the Canadian Psychological Association, and the NZ Psychological Society) have recognized cultural diversity as an important element of service users' experiences and lived reality and have developed practices and statements to affirm cultural respect as an ethical and professional expectation from their membership. However, a recent survey in New Zealand stands out as an example of the concerns Indigenous populations (Māori and Pasifika) have about the globalized model of evidence-based interventions built on a narrow, colonized view of evidence not aligning with Indigenous worldviews and undermining innovation (70). Plessas, McCormack and Kafantaris have discussed how the science of ABA can become responsive to Māori culture and how behavior analysts can make such contributions on all levels (practitioners, service providers and institutions) (30). A possible contribution of behavior analysts living and working in CANZUS countries could be to engage in collaborative initiatives with other fields of psychology in order to apply the technology of behavior to already constructed models of indigenous health and well-being (e.g., Te Whare Tapa Whā, Te Wheke, etc.), thus promoting a paradigm shift. Actions such as the new Code of Ethics for Behavior Analysts (71) effective from January 2022, make an explicit reference to our obligation to engage in activities that promote diverse therapeutic relationships. This requirement aligns with our three themes of connection, innovation and reflective practice. The more we explore barriers and facilitators of access to psychological services for behavior change, the more opportunities we find to improve the quality of life of our people.

This scoping review is part of a larger investigation, and will inform the development of a survey to answer two main questions: what are the barriers and facilitators, and what tools and practices exist in psychology (with a behavioral component) for an effective and equitable service delivery for Indigenous populations. Yet, the findings of this review can already inform practitioners on the importance of connecting, innovative and reflective practices when working with Indigenous clients.

### Strengths and Limitations

Although we used a comprehensive search strategy, it is possible that not all relevant papers were found. The reviewed literature did not always provide detailed information. For example, the Indigenous populations' socioeconomic status often was not explicitly defined. Further, there was heterogeneity of intervention designs and we could not assess the procedural integrity of the analyzed studies. This review was also limited in that we restricted our search to Indigenous populations in CANZUS nations only. New Zealand Māori do share similar histories of colonization with other CANZUS Indigenous Nations, though their experiences may have been different. Thus, our findings may be limited in their applicability to Indigenous populations in other countries (such as Indigenous populations in South America or Southeast Asia).

However, this review still allows for a broader view of the barriers and facilitators to psychological services for Indigenous individuals by including a large number of studies with multiple viewpoints. Parent training and adapted CBT were the two primary interventions examined in the literature reviewed, begging the question why cultural variables have not been examined in the context of other interventions. Though this is limiting the analysis to a narrow array of psychological services, the studies reviewed still provide us with useful information about a diversity of cultural adaptation efforts. This first review targeting psychological services with a behavioral component may unlock additional avenues to understanding some key elements of cultural adaptations enhancing the goal of behavior change.

## Conclusion

The findings of this scoping review suggest that connection, innovation and reflective practice are key to creating opportunities for effective and equitable services (either at a practitioner's level or that of an organization or institution). Our analysis suggests that our own actions determine the level of success we can attain in bringing together our practice and the recipients of treatment (connection), showing flexibility and persistence in finding solutions (innovation) and examining the role of our behavior in reaching our collaborative therapeutic goals (reflection). This information may contribute to building contemporary practices suitable for all and may thus assist therapists to broaden their scope of practice.

## Author Contributions

All authors contributed to the conception and design of the study. AP (Greek female psychologist, behavior analyst and researcher) wrote the first draft of the manuscript that was reviewed by all authors. AT (Indigenous clinical psychologist and academic) provided input into the cultural content and context of this research. AP, MB (Indigenous female research assistant), JM (NZ European female researcher in public health and psychology) participated in data extraction, while all authors contributed to data analysis (incl. AA-German female academic, supervisor and behavior analyst and ONM-Russian male academic and researcher). All authors approved the final version for submission and agreed to be accountable for all aspects of this work.

## Funding

This review was part of a larger investigation: Cultural Adaptation of Behavior-Analytic Services in Aotearoa funded by a University of Waikato Strategic Research Fund, 2020 Project Grant (108070).

## Conflict of Interest

The authors declare that the research was conducted in the absence of any commercial or financial relationships that could be construed as a potential conflict of interest.

## Publisher's Note

All claims expressed in this article are solely those of the authors and do not necessarily represent those of their affiliated organizations, or those of the publisher, the editors and the reviewers. Any product that may be evaluated in this article, or claim that may be made by its manufacturer, is not guaranteed or endorsed by the publisher.

## References

[1] Foxx RM. Applied behavior analysis treatment of autism: the state of the art. Child Adolesc Psychiatr Clin N Am. (2008) 17:821–34. 10.1016/j.chc.2008.06.007 18775372

[2] Dillenburger K Keenan M. None of the As in ABA stand for autism: dispelling the myths. J Intellect Dev Disabil. (2009) 34:193–5. 10.1080/13668250902845244 19404840

[3] Zarcone J Brodhead M Tarbox J. Beyond a call to action: an introduction to the special issue on diversity and equity in the practice of behavior analysis. Behav Anal Pract. (2019) 12:741–2. 10.1007/s40617-019-00390-1 31976284 PMC6834795

[4] Skinner BF. Selection by consequences. Science. (1981) 213:501–4. 10.1126/science.7244649 7244649

[5] Fong EH. Standards for culturally sensitive practice of applied behavior analysis. In: Conners BM, editor. Multiculturalism and Diversity in Applied Behavior Analysis. Oxford: Routledge (2020). p. 19–27.

[6] Brodhead MT Durán L Bloom SE. Cultural and linguistic diversity in recent verbal behavior research on individuals with disabilities: a review and implications for research and practice. Anal Verbal Behav. (2014) 30:75–86. 10.1007/s40616-014-0009-8 27274974 PMC4883540

[7] Sivaraman M Fahmie TA. A systematic review of cultural adaptations in the global application of ABA-based telehealth services. J Appl Behav Anal. (2020) 53:1838–55. 10.1002/jaba.763 32954539

[8] Beaulieu L Addington J Almeida D. Behavior analysts' training and practices regarding cultural diversity: the case for culturally competent care. Behav Anal Prac. (2019) 12:557–75. 10.1007/s40617-018-00313-6 31976264 PMC6743533

[9] Skinner BF. Science and Human Behavior. Oxford: Macmillan (1953). p. 461.

[10] Skinner BF. Beyond Freedom and Dignity. New York, NY: Knopf (1971). p. 240.

[11] Fong EH Catagnus RM Brodhead MT Quigley S Field S. Developing the cultural awareness skills of behavior analysts. Behav Anal Prac. (2016) 9:84–94. 10.1007/s40617-016-0111-6 27606242 PMC4788642

[12] Fong EH Ficklin S Lee HY. Increasing cultural understanding and diversity in applied behavior analysis. Behav Anal Res Prac. (2017) 17:103–13. 10.1037/bar0000076

[13] Fong EH Tanaka S. Multicultural alliance of behavior analysis standards for cultural competence in behavior analysis. J Behav Consult Ther. (2013) 8:17–9. 10.1037/h0100970

[14] Ministry of Health. Achieving Equity in Health Outcomes: Highlights of Important National and International Papers. (2018). Available online at: https://www.health.govt.nz/system/files/documents/publications/achieving-equity-in-health-outcomes-important-paper-highlights-nov18_1.pdf (accessed September 2, 2020).

[15] McIntyre C Harris MG Baxter AJ Leske S Diminic S Gone JP. Assessing service use for mental health by Indigenous populations in Australia, Canada, New Zealand and the United States of America: a rapid review of population surveys. Health Res Pol Syst. (2017) 15:67–84. 10.1186/s12961-017-0233-5 28778208 PMC5544983

[16] Health Quality and Safety Commision New Zealand. He matapihi ki te kounga o ngā manaakitanga ā hauora o Aotearoa/A window on the quality of Aotearoa New Zealand's health care 2019. (2019). Available online at: https://www.hqsc.govt.nz/assets/Health-Quality-Evaluation/PR/Window_2019_web_final.pdf (accessed December 12, 2020).

[17] Nguyen CT Krakowiak P Hansen R Hertz-Picciotto I Angkustsiri K. Sociodemographic disparities in intervention service utilization in families of children with autism spectrum disorder. J Autism Dev Disord. (2016) 46:3729–38. 10.1007/s10803-016-2913-3 27639855 PMC5112120

[18] Berry JW. Acculturation and psychological adaptation: a conceptual overview. In: Berry JW Annis RC, editors. Ethnic Psychology Research and Practice with Immigrants, Refugees, Native Peoples, Ethnic Groups and Sojourners. Amsterdam: Swets & Zeitlinger (1987). p. 41–52.

[19] Berry JW. Psychology of acculturation. In: Goldberger NR Veroff JB, editor. The Culture and Psychology Reader. New York, NY: New York University Press. (Reprinted from Berman JJ, editor. *Nebraska Symposium on Motivation: Cross-Culltural Perspectives*. The University of Nebraska Press, 1989) (1995). p. 457–88.

[20] Ogbu JU. Origins of human competence: a cultural-ecological perspective. In: Goldberger NR Veroff JB, editor. The Culture and Psychology Reader. New York, NY: New York University Press (1995). p. 245–75.

[21] Anderson A. Issues of migration. In: Hamilton R Moore D, editors. Educational Interventions for Refugee Children. London: Routledge Falmer (2004). p. 64–82.

[22] Smith T Williams P. Bergin and Garfield's handbook of psychotherapy and behavior change. In: Lambert MJ, editor. Behavioral Medicine and Clinical Health Psychology. 5th ed. Hoboken, NJ: Wiley (2004). p. 690–735.

[23] Bennett ST Flett RA Babbage DR. Considerations for culturally responsive cognitive-behavioural therapy for Māori with depression. J Pac Rim Psychol. (2016) e8:1–11. 10.1017/prp.2016.5

[24] Hinton DE Patel A. Cultural adaptations of cognitive behavioral therapy. Psychiatr Clin North Am. (2017) 40:701–14. 10.1016/j.psc.2017.08.006 29080595

[25] Castro FG Barrera M Holleran Steiker LK. Issues and challenges in the design of culturally adapted evidence-based interventions. Annu Rev Clin Psychol. (2010) 6:213–39. 10.1146/annurev-clinpsy-033109-132032 20192800 PMC4262835

[26] Chowdhary N Jotheeswaran AT Nadkarni A Hollon SD King M Jordans MJD. The methods and outcomes of cultural adaptations of psychological treatments for depressive disorders: a systematic review. Psychol Med. (2014) 44:1131–46. 10.1017/S0033291713001785 23866176 PMC3943384

[27] Degnan A Baker S Edge D Nottidge W Noke M Press CJ. The nature and efficacy of culturally-adapted psychosocial interventions for schizophrenia: a systematic review and meta-analysis. Psychol Med. (2018) 48:714–27. 10.1017/S0033291717002264 28830574

[28] Dennison A Lund EM Brodhead MT Mejia L Armenta A Leal J. Delivering home-supported applied behavior analysis therapies to culturally and linguistically diverse families. Behav Anal Pract. (2019) 12:887–98. 10.1007/s40617-019-00374-1 31976301 PMC6834806

[29] Cihon TM Mattaini MA. Editorial: emerging cultural and behavioral systems science. Perspect Behav Sci. (2019) 42:699–711. 10.1007/s40614-019-00237-8 31976456 PMC6901637

[30] Plessas A McCormack J Kafantaris I. The potential role of applied behavior analysis in the cultural environment of Māori mental health. Behav Anal Prac. (2019) 12:854–68. 10.1007/s40617-019-00359-0 31976298 PMC6834803

[31] JBI. Joanna Briggs Institute reviewer's manual 2015: methodology for JBI scoping reviews. In: Joanna Briggs Institute (JBI), editor. JBI Manual for Evidence Synthesis. Adelaide, SA: The University of Adelaide (2015).

[32] Ministry of Health. Ministry of Health, Manatu Hauora (n.d). Available online at: https://www.health.govt.nz/ (accessed October 13, 2020).

[33] Braun V Clarke V. Reflecting on reflexive thematic analysis. Qual Res Sport Exerc Health. (2019) 11:589–97. 10.1080/2159676X.2019.1628806

[34] Boyatzis RE. Transforming Qualitative Information: Thematic Analysis and Code Development. Thousand Oaks, CA: Sage Publications (1998). p. 200.

[35] Andersson E MCLLduff C Turner K Thomas S Devies J Elliott E. Jandu Yani U ‘for all families' triple p-positive parenting program in remote Australian aboriginal communities: a study protocol for a community intervention trial. BMJ Open. (2019) 9. 10.1136/bmjopen-2019-032559 31601605 PMC6797340

[36] Bennett ST Flett RA Babbage DR. Culturally adapted cognitive behaviour therapy for Māori with major depression. Cogn Behav Ther. (2014) e20:1–16. 10.1017/S1754470X14000233

[37] Bennett-Levy J Wilson S Nelson J Stirling J Ryan K Rotumah D. Can CBT be effective for aboriginal Australians? Perspectives of Aboriginal practitioners trained in CBT. Aust Psychol. (2014) 49:1–7. 10.1111/ap.12025

[38] BigFoot DS Schmidt SR. Honoring children, mending the circle: cultural adaptation of trauma-focused cognitive-behavioral therapy for American Indian and Alaska Native children. J Clin Psychol. (2010) 66:847–56. 10.1002/jclp.20707 20549679

[39] Chomos JC Evans WP Bolan M Merritt L Meyer A Novins DK. Using single-case designs to evaluate components of tribal home-visitation programs. Infant Ment Health J. (2018) 39:335–46. 10.1002/imhj.21712 29726592

[40] Cope V. Ngā tau miharo-Incredible Years parent programme-empowering whānau through manaakitanga. Te Kura Nui o Waipareira. (2018) 7:9–15.

[41] Gameon JA Skewes MC. A systematic review of trauma interventions in native communities. Am J Community Psychol. (2020) 65:223–41. 10.1002/ajcp.12396 31518009 PMC7243818

[42] Gone JP Trimble JE. American Indian and Alaska Native mental health: Diverse perspectives on enduring disparities*Annu Rev Clin Psychol*. (2012) 8:131–60. 10.1146/annurev-clinpsy-032511-143127 22149479

[43] Goodkind JR Ross-Toledo K John S Hall JL Ross L Freeland L. Promoting healing and restoring trust: policy recommendations for improving behavioral health care for American Indian/Alaska Native adolescents. Am J Community Psychol. (2010) 46:386–94. 10.1007/s10464-010-9347-4 20857331 PMC3041509

[44] Gutierrez L Chadwick N Wanamaker KA. Culturally relevant programming versus the status quo: a meta-analytic review of the effectiveness of treatment of indigenous offenders. Can J Criminol Crim Justice. (2018) 60:321–53. 10.3138/cjccj.2017-0020.r2

[45] Haozous EA Doorenbos AZ Stoner S. Pain management experiences and the acceptability of cognitive behavioral strategies among American Indians and Alaska Natives. J Transcult Nurs. (2016) 27:233–40. 10.1177/1043659614558454 25403169 PMC4433858

[46] Hart LM Jorm AF Kanowski LG Kelly CM Langlands RL. Mental health first aid for Indigenous Australians: using Delphi consensus studies to develop guidelines for culturally appropriate responses to mental health problems. BMC Psychiatry. (2009) 9:47. 10.1186/1471-244X-9-47 19646284 PMC2729076

[47] Hatcher S Coupe N Wikiriwhi K Durie SM Pillai A. Te Ira Tangata: a Zelen randomised controlled trial of a culturally informed treatment compared to treatment as usual in Māori who present to hospital after self-harm. Soc Psychiatry Psychiatr Epidemiol. (2016) 51:885–94. 10.1007/s00127-016-1194-7 26956679

[48] Johnson JL Cameron MC. Barriers to providing effective mental health services to American Indians. Ment Health Serv Res. (2001) 3:215–23. 10.1023/A:1013129131627 11859967

[49] Keown LJ Sanders MR Franke N Shepherd M. Te Whānau Pou Toru: a randomized controlled trial (RCT) of a culturally adapted low-intensity variant of the Triple P-Positive Parenting Program for Indigenous Māori families in New Zealand. Prev Sci. (2018) 19:954–65. 10.1007/s11121-018-0886-5 29564752

[50] Kilcullen M Swinbourne A Cadet-James Y. Aboriginal and Torres strait islander health and well-being: implications for a cognitive behavioural therapy framework. Aust Psychol. (2016) 52:453–62. 10.1111/ap.12159

[51] Kulis SS Tsethlikai M Harthun ML Hibbeler PK Ayers SL Deschine Parkhurst N. Parenting in 2 worlds: effects of a culturally grounded parenting intervention for urban American Indians on participant cultural engagement. Cult Divers Ethn Min Psych. (2019) 26:237–446. 10.1037/cdp0000315 31886683 PMC7326650

[52] Lewis ME Myhra LL. Integrated care with indigenous populations: considering the role of health care systems in health disparities. J Health Care Poor Underserved. (2018) 29:1083–107. 10.1353/hpu.2018.0081 30122685

[53] Lints-Martindale AC Carlson AA Goodwin SL Thompson SN. Putting recommendations into practice: improving psychological services in rural and northern Canada. Can Psychol. (2018) 59:323–31. 10.1037/cap0000158

[54] Lipsham M. Āta as an innovative method and practice tool in supervision. Aotearoa NZ Soc Work. (2012) 24:31. 10.11157/anzswj-vol24iss3-4id122

[55] Mathieson F Mihaere K Collings S Dowell A Stanley J. Maori cultural adaptation of a brief mental health intervention in primary care. J Prim Health Care. (2012) 4:231–8. 10.1071/HC12231 22946072

[56] McCabe GH. The healing path: a culture and community-derived indigenous therapy model. Psychother Theor Res Pract Train. (2007) 44:148–60. 10.1037/0033-3204.44.2.148 22122207

[57] McIntosh K Moniz C Craft CB Golby R Steinwand-Deschambeault T. Implementing school-wide positive behavioural interventions and supports to better meet the needs of indigenous students. Can J Sch Psychol. (2014) 29:236–57. 10.1177/0829573514542217

[58] Morsette A van den Pol R Schuldberg D Swaney G Stolle D. Cognitive behavioral treatment for trauma symptoms in American Indian youth: preliminary findings and issues in evidence-based practice and reservation culture. Adv Sch Ment Health Promot. (2012) 5:51–62. 10.1080/1754730X.2012.664865

[59] Pina AA Polo AJ Huey SJ. Evidence-based psychosocial interventions for ethnic minority youth: the 10-year update. J Clin Child Adolesc Psychol. (2019) 48:179–202. 10.1080/15374416.2019.1567350 30746965

[60] Rangihuna D Kopua M Tipene-Leach D. Mahi a Atua: a pathway forward for Māori mental health? N Z Med J. (2018) 131:79–83. 29518802

[61] Robinson G Tyler W Jones Y Silburn S Zubrick SR. Context, diversity and engagement: early intervention with Australian aboriginal families in urban and remote contexts. Child Soc. (2012) 26:343–55. 10.1111/j.1099-0860.2010.00353.x

[62] Sexton E Starr E Fawcett M. Identifying best practice principles in working with the Inupiat Eskimo: building trust and beyond. Int J Adv Couns. (2005) 27:513–22. 10.007/s10447-005-8488-x

[63] Snowden LR Yamada AM. Cultural differences in access to care. Annu Rev Clin Psychol. (2005) 1:143–66. 10.1146/annurev.clinpsy.1.102803.143846 17716085

[64] Stead R. A discussion of the principle of cultural responsiveness: from research to practice and from history to today. Kairaranga. (2014) 15:5–10.

[65] Stock C Mares S Robinson G. Working together in a good way: relationships between local Indigenous and fly-in workers delivering a parent-child programme in remote Aboriginal communities. Int Soc Work. (2019) 62:48–61. 10.1177/0020872817710545

[66] Tauai E Richards R Kokaua J. Is Pacific language ability protective of prevalence of mental disorders among Pacific peoples in New Zealand? Pac Health Dialog. (2018) 21:10–6. 10.26635/phd.2018.902

[67] Tsosie U Nannauck S Buchwald D Russo J Trusz SG Foy H. Staying connected: a feasibility study linking American Indian and Alaska Native trauma survivors to their tribal communities. Psychiatry. (2011) 74:349–61. 10.1521/psyc.2011.74.4.349 22168295 PMC3795506

[68] Turner KMT Sanders MR Hodge L. Issues in professional training to implement evidence-based parenting programs: the preferences of indigenous practitioners. Aust Psychol. (2014) 49:384–94. 10.1111/ap.12090

[69] Wagner B Fitzpatrick J Symons M Jirikowic T Cross D Latimer J. The development of a culturally appropriate school based intervention for Australian Aboriginal children living in remote communities: a formative evaluation of the Alert Program® intervention. Aust Occup Ther J. (2017) 64:243–52. 10.1111/1440-1630.12352 27966224

[70] Bullen P Deane KL Meissel K Bhatnagar S. What constitutes globalised evidence? Cultural tensions and critical reflections of the evidence-based movement in New Zealand. Int J Psychol. (2020) 55:16–25. 10.1002/ijop.12574 30779343

[71] Behavior Analyst Certification Board. Ethics Code for Behavior Analysts. Littleton: Littleton Publishing (2020). p. 19.

